# Richter-Type Spigelian Hernia Presenting With Small Bowel Obstruction: A Case Report

**DOI:** 10.7759/cureus.95248

**Published:** 2025-10-23

**Authors:** Mohammed Jameel, Ahmad Alabood

**Affiliations:** 1 Emergency Department, East Lancashire Teaching Hospitals, Blackburn, GBR

**Keywords:** bowel obstruction, ct-abdomen and pelvis with contrast, richter hernia, spigelian hernia, surgey

## Abstract

Spigelian hernia is a rare ventral abdominal wall hernia occurring along the semilunar line (Spigelian fascia), accounting for approximately 1%-2% of all hernias. It often presents with non-specific abdominal pain and carries a high risk of bowel incarceration and strangulation. Because the hernia sac lies beneath muscle, a palpable mass may be absent, making diagnosis challenging without imaging.

We report a case of a previously healthy middle-aged man who awoke with sharp right lower quadrant pain. Physical exam revealed localized tenderness and a 2×4 cm mass that was deep, small, and obscured by overlying muscle in the right iliac fossa with audible bowel sounds. Imaging confirmed a right Spigelian hernia containing ileal loops with features of small bowel obstruction. An emergency combined laparoscopic and open hernia repair was performed; the incarcerated bowel was viable and reduced, and a mesh was placed to reinforce the defect.

The patient’s postoperative course was uneventful with a gradual return of bowel function and no complications. This case underscores the importance of high clinical suspicion and prompt imaging in diagnosing Spigelian hernias. Early surgical intervention in such cases can prevent strangulation and leads to favorable outcomes.

## Introduction

The term “Spigelian hernia” was first coined by a Flemish anatomist, Josef Klinkosch, to describe a defect in the semilunar line [1]. Accounting for 1%-2% of all hernias, Spigelian hernia (SpH) is the protrusion of preperitoneal fat, peritoneal sac, or organ(s) through a congenital or acquired defect in the spigelian aponeurosis (i.e., the aponeurosis of the transverse abdominal muscle limited by the linea semilunaris laterally and the lateral edge of the rectus muscle medially) [1, 2]. Almost 90% of all SpHs occur within the so-called “Spigelian belt” (a transverse band around the level of the semicircular line, typically below the umbilicus) [3]. SpH mainly affects the adult population, with a median age of 65 years at diagnosis [4,5]. SpH is more frequent in women, with a reported female-to-male ratio of 2:1 [4, 6]. In the initial stages of development, SpHs are often difficult to diagnose by physical examination because the hernia originates inferior to an intact external oblique aponeurosis [7]. Physical diagnosis correctly identifies SpHs approximately 50% of the time; thus, with the advances in radiological imaging, an increase has occurred in the number of SpH cases diagnosed in the past decade compared with previous decades. Both ultrasound and computed tomography are useful radiological adjuncts for diagnosing SpHs [7]. Once SpH is diagnosed, there is a need for surgical treatment because of the high risk for serious complications. Emergency surgery is estimated to be performed in 21%-33% of cases due to incarceration and strangulation [8-10]. We present a case of an incarcerated SpH in a previously healthy man, diagnosed and managed at the Royal Blackburn Hospital, to highlight the clinical presentation, radiologic findings, and successful surgical management of this condition.

## Case presentation

A 46-year-old man (previously healthy, with no prior surgeries) presented to the emergency department with an acute-onset abdominal pain. The pain began abruptly at around 11:00 PM while he was preparing for bed. It was sharp and severe (8/10 intensity), localized to the right lower quadrant (RLQ) with no radiation. He reported no nausea or vomiting, and his appetite remained intact. He attempted over-the-counter analgesics for pain relief, which provided only temporary improvement. As the pain recurred with greater intensity and persisted into the night, the patient and his wife sought medical attention. On evaluation in the hospital, his vital signs were largely within normal limits (afebrile at 36.2°C, heart rate 83/min, blood pressure 157/104 mmHg, respirations 18/min, oxygen saturation (SpO₂) 96% on room air). He appeared alert and oriented, in moderate discomfort from the abdominal pain. Abdominal examination revealed tenderness focused in the RLQ. Notably, a firm, well defined mass ~2×4 cm was palpable in the right iliac fossa lateral to the rectus muscle. The mass was tender and irreducible on exam. There was no overlying skin change, and it moved slightly with respiration but independently from the overlying skin. The area was tympanic on percussion and the bowel sounds were audible over the mass on auscultation. There was mild guarding in the RLQ, but no rebound tenderness or rigidity, and bowel sounds were present in all quadrants. No other abdominal masses or hernias were evident on exam. The remainder of the systemic examination was unremarkable.

Bedside ultrasound was performed focusing on the area of the RLQ mass. A loop of bowel was visualized within the abdominal wall defect with active peristalsis, confirming the mass to be an entrapped segment of intestine (consistent with a SpH).

Full blood count and metabolic panel were essentially normal: white cell count was 7.4×10^9/L with normal neutrophils count of 3.2×10/L, hemoglobin 150 g/L, and C-reactive protein <3.3 mg/L. Venous blood gas analysis showed a mild elevated partial pressure of carbon dioxide (pCO₂) of 7.0 kPa with normal lactate (2.6 mmol/L), which was not clinically significant. All other blood chemistry values were within normal limits.

Given the physical exam and ultrasound findings suggesting an incarcerated SpH, an urgent contrast-enhanced CT scan of the abdomen and pelvis was obtained for definitive diagnosis and to assess for bowel obstruction.

**Figure 1:**
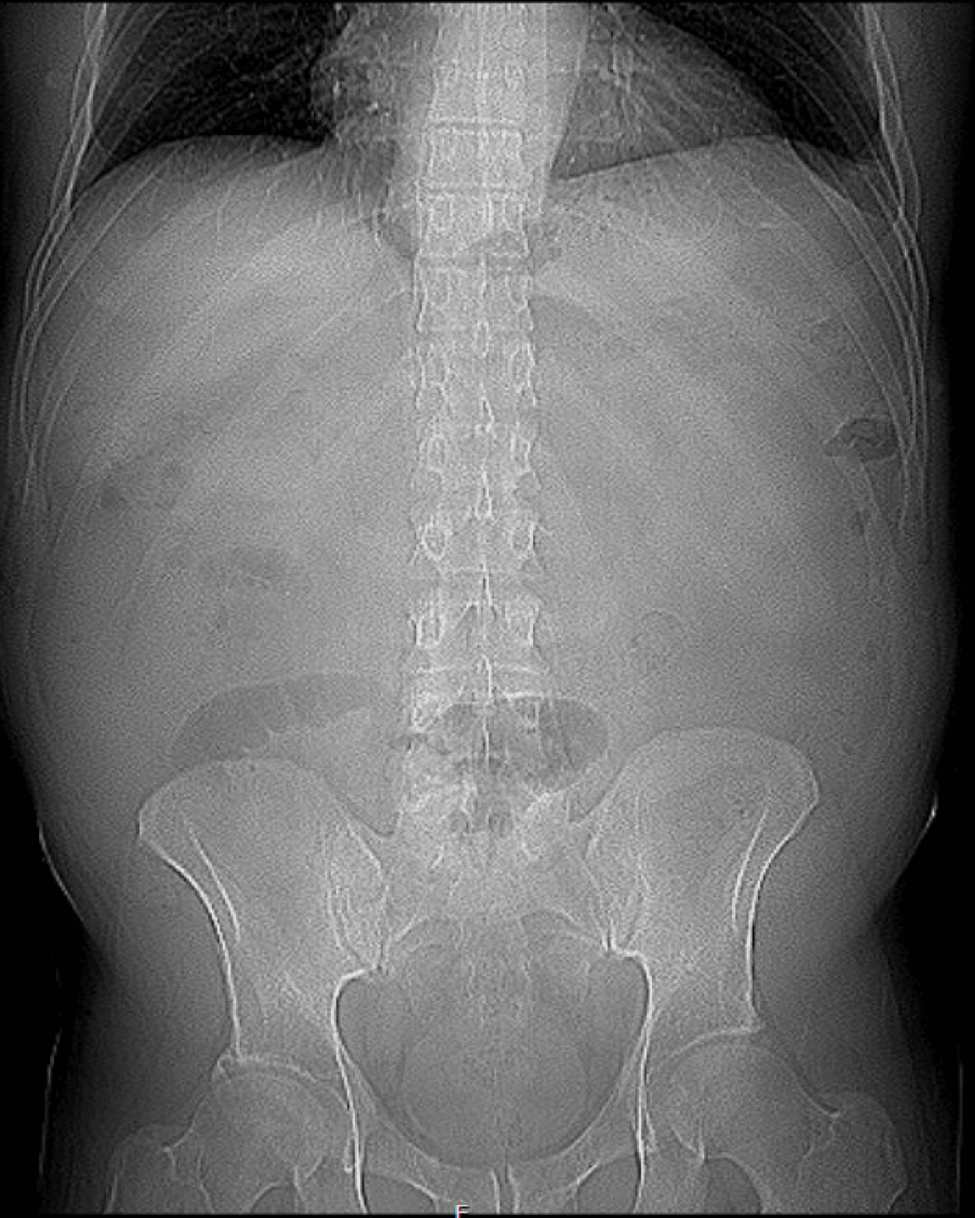
The abdominal X-ray demonstrates nonspecific bowel gas distribution without radiographic evidence of obstruction or perforation. No hernia sac is visualized, which is consistent with the known limitations of plain films in detecting Spigelian hernias. Further evaluation with contrast-enhanced CT is recommended for definitive diagnosis and assessment.

**Figure 2:**
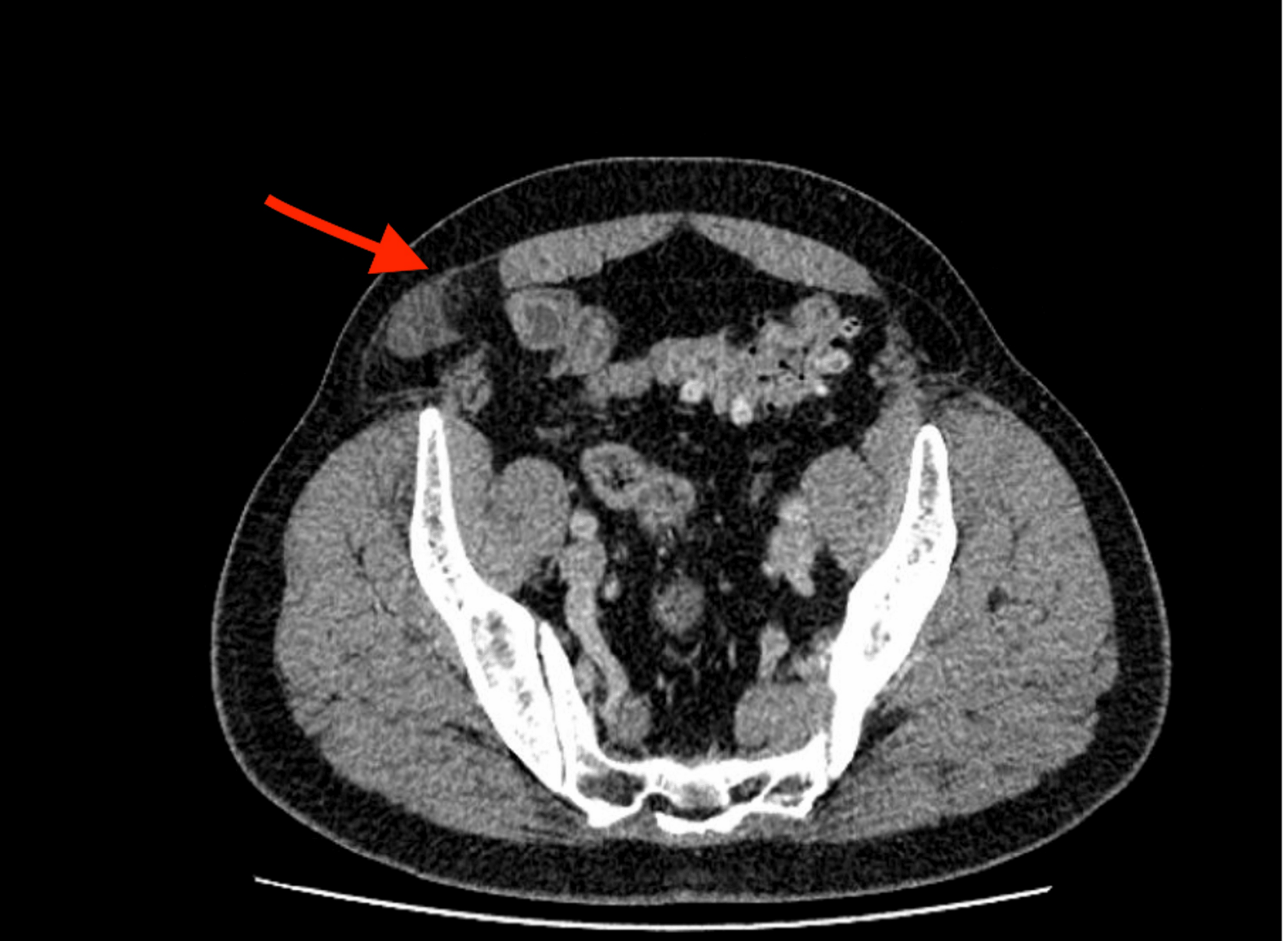
Axial CT scan image showing a Spigelian hernia (red arrow) at the right lateral abdominal wall. A loop of small bowel is seen protruding through a defect in the Spigelian fascia (semilunar line), with adjacent mesenteric fat. Such imaging confirms the presence of an incarcerated Spigelian hernia and often demonstrates any resulting bowel obstruction.

**Figure 3:**
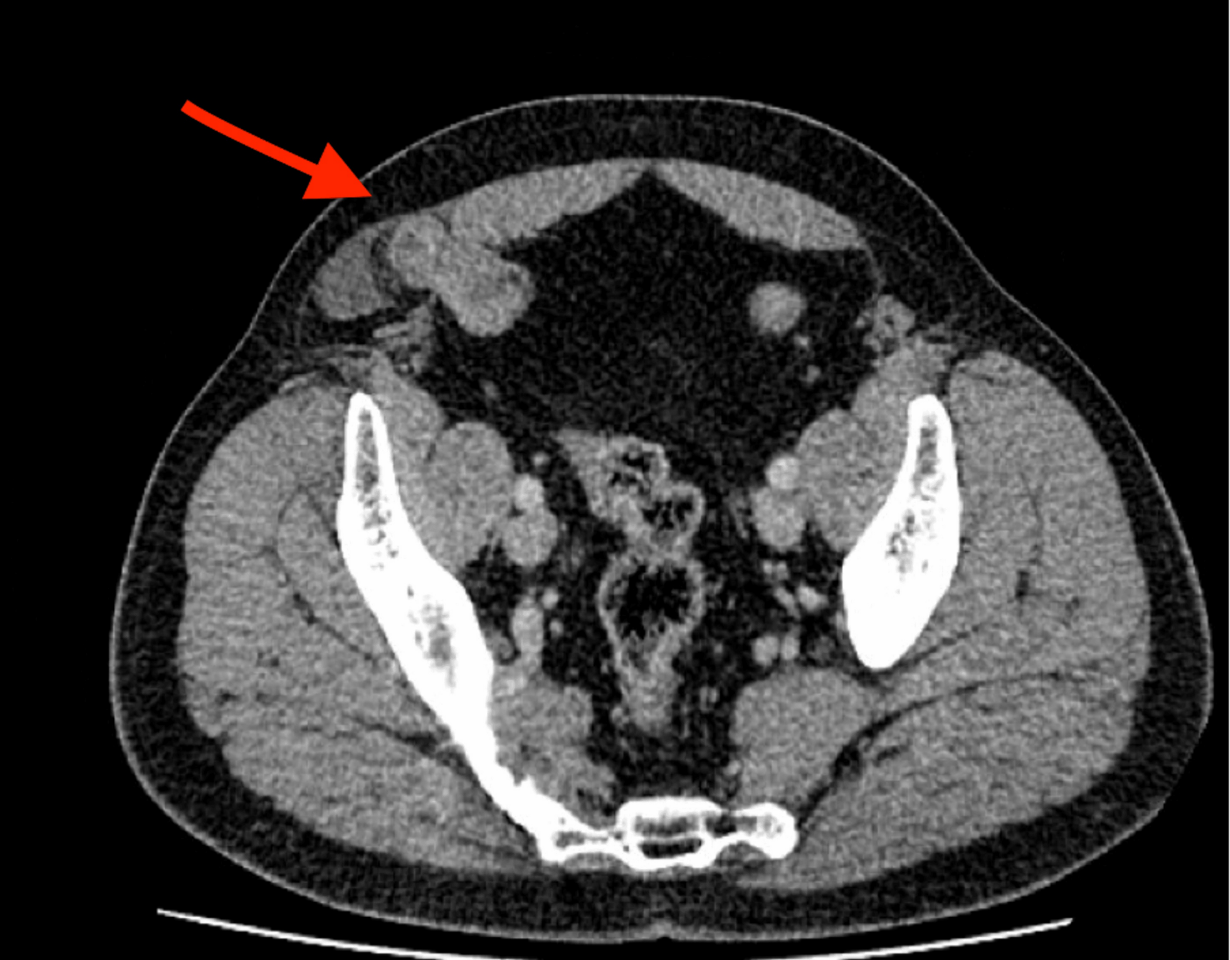
Axial contrast-enhanced CT of the abdomen demonstrates a right-sided abdominal wall defect through the Spigelian fascia, lateral to the rectus abdominis muscle (red arrow), containing a loop of small bowel. This finding is consistent with a right-sided Spigelian hernia.

**Figure 4:**
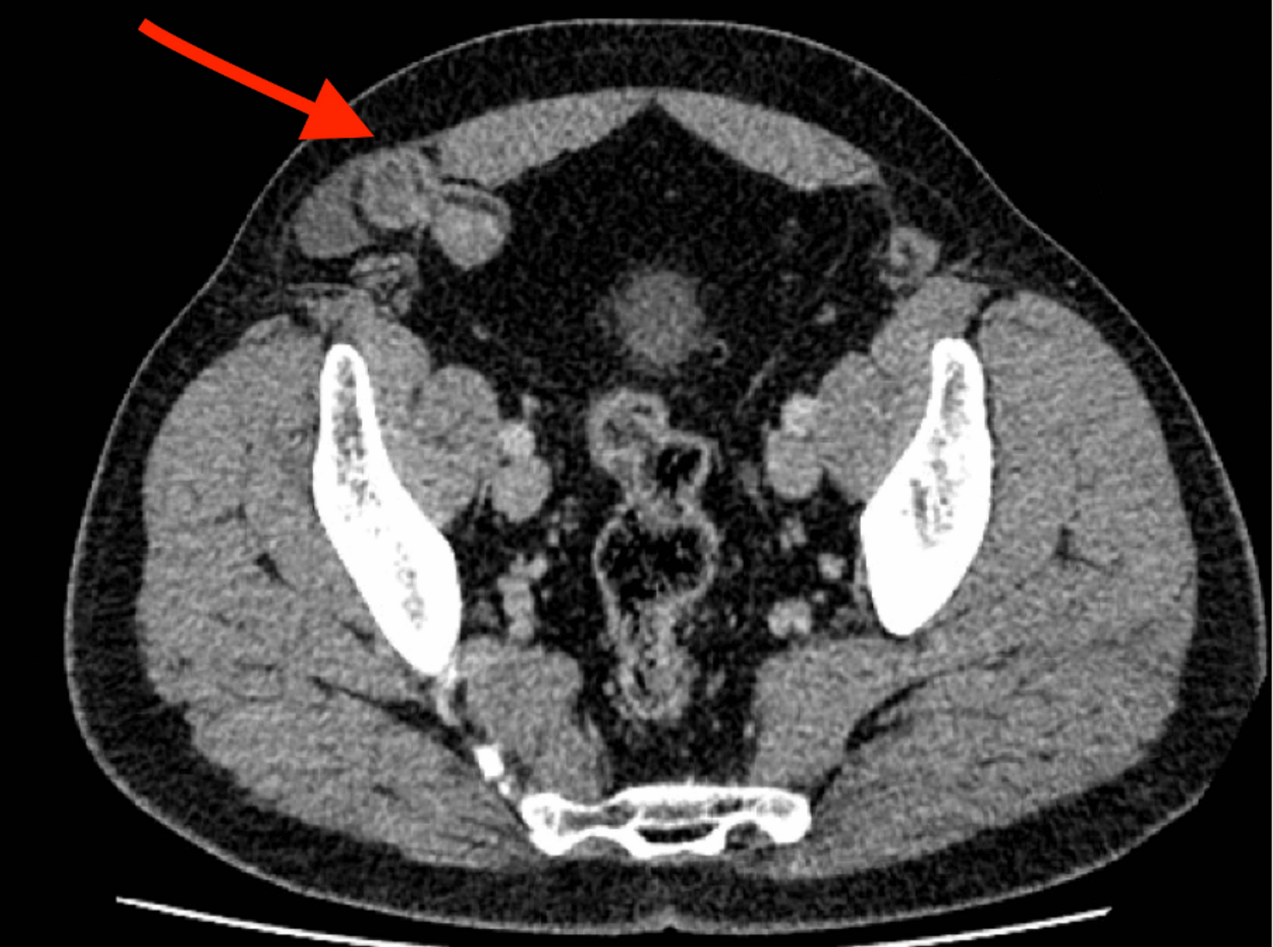
Axial contrast-enhanced CT of the abdomen demonstrates a right-sided abdominal wall defect through the Spigelian fascia, lateral to the rectus abdominis muscle (red arrow), containing a loop of small bowel. This finding is consistent with a right-sided Spigelian hernia.

The CT revealed a loop of small bowel (ileum) herniating through a defect in the right anterior abdominal wall, lateral to the rectus abdominis muscle. The defect was in the Spigelian fascia (right semilunar line) approximately 10 cm proximal to the ileocecal valve. The herniated bowel appeared pinched at the fascial defect. Upstream of this point, small bowel loops were mildly dilated and showed air-fluid levels, consistent with a partial small bowel obstruction; bowel loops distal to the hernia were decompressed. These findings confirmed the diagnosis of an incarcerated right Spigelian hernia causing small bowel obstruction [11]. The CT scan showed no free intraperitoneal air or free fluid and, importantly, there were no signs of perforation or abscess. Figures 1-4 illustrate an X-ray and serial CT images presented in a cranio-caudal sequence sequence, demonstrating the gradual appearance and anatomical progression of the hernia. The site of herniation is marked by the red arrow and is situated on the right side, as visualized in the axial planes. 

After imaging confirmation, the patient was kept nil per mouth (NPO) and started on intravenous fluids.

Surgical consultation was obtained emergently. Given the evidence of incarceration (and partial obstruction) but absence of peritonitis or strangulation signs, the surgical team planned an urgent operative repair. The patient was taken to the operating theatre on the next available list (within 12 hours of presentation).

Intraoperative findings

Under general anesthesia, diagnostic laparoscopy was first performed. Trocars were placed in the left abdomen (one 10 mm port at the left midclavicular line, level of the umbilicus, and two 5 mm ports in the left upper and lower quadrants). Upon insufflation and laparoscopic evaluation, the site of the hernia in the RLQ was identified from inside the abdomen. A segment of ileum ~10 cm proximal to the ileocecal junction was found to have been incarcerated in the Spigelian defect. By the time of surgery, this segment had spontaneously reduced back into the abdominal cavity (likely from patient positioning and pneumoperitoneum), but signs of local trauma to the bowel wall were present. Notably, this was a Richter-type hernia, meaning only the antimesenteric border of the bowel circumference had been entrapped in the hernia. The affected ileal segment showed mild congestion on the antimesenteric side but no full-thickness ischemia. Indocyanine green (ICG) fluorescence angiography was utilized laparoscopically to assess bowel perfusion: the involved loop demonstrated prompt uptake of ICG dye, confirming intact blood supply. Therefore, no bowel resection was necessary as the intestine was viable. Given the emergent setting and to facilitate a durable repair of the fascial defect, the surgeons elected to proceed with an open hernia repair at the site of the SpH. A small transverse incision (~5 cm) was made directly over the RLQ hernia site. The external oblique aponeurosis was incised, and dissection carried down to the hernia sac. The hernia sac (containing the previously incarcerated bowel) was identified between the layers of the abdominal wall and carefully dissected free from surrounding tissues. A preperitoneal plane was developed around the defect, separating the peritoneum from the underside of the abdominal musculature for a few centimeters in all directions. The hernia orifice measured approximately 2×3 cm and was closed primarily with interrupted 2-0 polypropylene (Prolene) sutures, which were chosen for their high tensile strength, minimal tissue reactivity, and long-term durability, ensuring a secure fascial repair. A synthetic composite mesh (~8 cm size) was then placed in the preperitoneal space to cover the defect, extending well beyond the margins of the hernia opening. The composite mesh was selected to provide durable reinforcement while minimizing the risk of adhesions, given its dual-surface design suitable for the preperitoneal location. The mesh was secured with several interrupted sutures to the undersurface of the internal oblique/transversus layer (anchors placed superiorly, inferiorly, medially, and laterally), reinforcing the area. The fascial edges of the internal oblique were also approximated with additional sutures over the mesh, and the external oblique aponeurosis was closed over top. The incision was closed in layers, and skin was sutured with an absorbable subcuticular stitch. There were no intraoperative complications. The combined laparoscopic and open approach achieved both a thorough exploration (to evaluate the bowel) and a solid repair of the hernia defect with mesh.

Treatment and follow-up

The patient was transferred to the surgical ward for recovery. Standard deep vein thrombosis prophylaxis was given (graduated compression stockings and low-molecular-weight heparin) and adequate analgesia provided for pain control. He was managed initially with the head of bed elevated and kept NPO until return of bowel function. 

The nasogastric tube placed intraoperatively was removed the next morning. 

By postoperative day 1, the abdominal pain was markedly improved and the patient had no significant abdominal distension. Bowel sounds were present and he passed flatus. A clear liquid diet was started and advanced gradually as tolerated; he transitioned to soft solids by day 2 without incident. 

Serial abdominal examinations showed a soft, nondistended abdomen with well healing incision and no signs of recurrent hernia or ischemia. Given the short-term duration of follow-up, long-term outcomes such as recurrence rate or chronic pain cannot yet be conclusively assessed. Continued surveillance is planned at three, six and 12 months postoperatively.

Written informed consent was obtained from the patient for publication of this case report and the accompanying image. The patient understood that no personal identifying information would be disclosed and agreed to the publication of the clinical details and findings.

## Discussion

This case presents a classic case of an incarcerated SpH causing small bowel obstruction. It also highlights several important aspects in the diagnosis and management of this condition. SpH is relatively rare and its diagnosis can be challenging. Patients may present with acute abdominal pain, but because the hernia protrusion is often interparietal, visible swelling on the abdominal wall is often absent or subtle [12].

In our patient, careful physical examination did reveal a small tender mass in the RLQ with tympanic percussion and bowel sounds on auscultation, which raised suspicion for an abdominal wall hernia containing bowel. However, such clinical findings can be elusive, and as the literature suggests, up to half of SpHs may be missed or misdiagnosed prior to imaging [13]. In one review, only about 50% of cases were correctly identified preoperatively without advanced imaging. Ultrasound can aid in diagnosis and was useful in this case to confirm bowel loops in the abdominal wall, but CT scanning remains the gold standard for diagnosis [13,14]. Cross-sectional imaging not only localizes the hernia defect but also delineates the hernia contents and any complications (obstruction, strangulation), guiding urgent management [14]. Indeed, CT in our case was crucial in confirming the SpH and small bowel obstruction, allowing timely surgical intervention. An interesting aspect of this case was the Richter-type hernia component.

A Richter’s hernia is a variant where only part of the circumference of the bowel (usually the antimesenteric border) is entrapped in the hernia defect, rather than the entire lumen [15]. In a Richter hernia, strangulation of the bowel wall can occur without leading to a complete luminal obstruction. This means patients might not have classic signs of high intestinal obstruction. In our patient, the bowel loop was partially incarcerated (antimesenteric side trapped), which resulted in a partial, sub-occlusive obstruction (some bowel dilation on CT with continued distal gas passage). This correlates with the known fact that Richter’s hernias seldom cause full bowel obstruction since only one side of the intestine is compromised [15,16]. In the context of a SpH, Richter-type presentation is uncommon but has been reported [17]. The simultaneous occurrence of a Richter hernia through a Spigelian defect is considered rare in the literature, yet our case demonstrates that it can occur and must be recognized.

The surgical team’s use of ICG fluorescence in this case is noteworthy, as it provided real-time assessment of bowel perfusion. This adjunct helped confirm that the incarcerated bowel, though congested, had viable blood flow after reduction, obviating the need for resection. Newer intraoperative imaging techniques like ICG angiography can thus be valuable in guiding surgical decisions in borderline cases of strangulation [18]. The management of Spigelian hernia is invariably surgical. Given the high risk of incarceration and strangulation, once diagnosed, prompt operative repair is advised even for asymptomatic cases. Both open and laparoscopic approaches are described for Spigelian hernioplasty, and each has its indications. In general, for uncomplicated SpHs (no bowel compromise), a primary laparoscopic repair is often preferred due to the advantages of minimal invasiveness and the ability to inspect both abdominal wall and intra-abdominal contents [19]. Laparoscopic techniques may include placing an intraperitoneal mesh or performing an extraperitoneal mesh repair, and they have shown low recurrence rates. However, if the defect is large (commonly >5 cm) or in an emergency setting with potentially compromised bowel, an open approach may be warranted [20]. In our case, the surgeons employed a hybrid approach: initial laparoscopy allowed thorough examination of the incarcerated bowel (and its successful reduction and viability assessment), while the definitive repair of the abdominal wall was done via a small open incision. This strategy combined the strengths of both methods - ensuring the safety of the bowel and a secure mesh repair of the defect. Tension-free mesh repair reduces recurrence risk, and in our patient, an 8-cm composite mesh was placed in the preperitoneal plane with excellent outcome.

The postoperative course of our patient highlights that favourable prognosis is very much achievable with timely management of SpH.

Finally, this case highlights the importance of considering abdominal wall hernias in the differential diagnosis of acute abdomen, especially for pain localized to the lateral abdominal regions. SpH, while rare, is a surgically correctable cause of acute abdominal pain and bowel obstruction that should not be missed.

## Conclusions

Even if rarely symptomatic, the Spigelian hernia is an entity to consider in the differential diagnosis of small bowel obstruction in a virgin abdomen and it requires a high index of suspicion to diagnose. This case report demonstrates that even in a previously healthy individual, acute onset of localized abdominal pain could be due to an incarcerated Spigelian hernia. Because clinical signs may be subtle or misleading, early use of imaging (particularly CT scan) is crucial to identify the hernia and associated complications. Timely surgical intervention is essential given the hernia’s tendency for incarceration and strangulation; such prompt management can avert serious outcomes like bowel necrosis and lead to excellent recovery. The successful outcome in our patient highlights that a combined laparoscopic and open surgical approach can be safe and effective, especially in emergency scenarios.
